# Rare multiple primary malignancies among surgical patients—a single surgical unit experience

**DOI:** 10.3332/ecancer.2014.438

**Published:** 2014-06-18

**Authors:** Nicola Carlomagno, Michele L Santangelo, Rossella Mastromarino, Armando Calogero, Concetta Dodaro, Andrea Renda

**Affiliations:** 1 General Surgery, Department of Advanced Biomedical Sciences, University of Naples Federico II, via S. Pansini, 80131 Naples, Italy,; 2 General Surgery and Transplant Unit, Department of Advanced Biomedical Sciences, University of Naples Federico II, via S. Pansini, 80131 Naples, Italy

**Keywords:** cancer treatment, follow-up, multiple primary malignancy (MPM)

## Abstract

**Background:**

A remarkable number of patients presents with multiple primary malignancies (MPM) over their lifetimes. In most cases inherited syndromes, iatrogenic, or viral factors are implicated, while in some cases it is not possible to ascertain a clear aetiopathogenesis.

**Methods:**

Starting from a series of 315 patients with MPM, we focused our attention on those with extremely infrequent combinations of tumours. We retrospectively analysed patients’ characteristics, type of first and second tumour and the interval between the two tumours. We made a comparison between our own data and data from surveillance, epidemiology, and end results cancer registries, the largest global series on this topic.

**Results:**

Six patients presented with unusual associations, namely, central nervous system (CNS)/colon, testis/stomach, colon/CNS, CNS/kidney, uterus/soft tissue, and bone/breast. The median age was 50.5 years at the diagnosis of second neoplasm and the male:female ratio was 1:1. All six patients underwent surgery for both tumours. The median interval between the first and the second tumour was 11.3 years (range 1–36 years). Five patients were given chemotherapy as adjuvant systemic treatment, and two of them with CNS tumours also received radiotherapy.

**Discussion:**

We analysed the behaviour of these rare tumours as first and second neoplasms. More frequent combinations and possible aetiological factors were evaluated.

**Conclusions:**

Follow-up for patients recovering from a first tumour must be strict, as there is the risk of developing MPM, even after a long time period. Advancement in biomolecular knowledge and cooperation among different specialists are strongly needed to reduce mortality related to MPM and to foresee their occurrence.

## Background

Since the end of the nineteenth century (1889), multiple primary malignancies (MPMs) have raised interest in the medical community. MPMs were defined as: (1) malignant tumours from the histopathology viewpoint, (2) topographic distinct without connection with submucosal or intra-epithelial alterations (skip metastasis), (3) leaving out a second tumour being a metastasis of the first one [1–6].

Moertel was the first, in 1961, to attempt to classify MPMs as simultaneous, synchronous, and metachronous, according to the interval between onset of the first and second tumour [2]. In 2009, we proposed a classification firstly based on the distinction between inherited forms and sporadic ones, which may be further divided according to their etiology: genetic, environmental, hormonal, immunological, iatrogenic (due to the anticancer treatments themselves), or viral [6]. Conversely, in some cases, a clear aetiopathogenesis is not recognisable at the best of our current biomedical knowledge, and in this case, we speak of ‘uncodified’ MPMs [6].

The number of patients diagnosed with MPM over a lifetime is increasing, and it is expected to grow further in the coming years. This observation might be explained by several factors, such as the increase in the incidence of cancer itself, the overall longer lifetime, the improvements in adjuvant treatments with accordingly longer disease-free interval, the accuracy of follow-up in neoplastic patients, and the relatively good prognosis of various tumours [4].

MPM population is very heterogeneous. For many patients, MPMs are attributable to inherited disorders [i.e., familial adenomatous polyposis (FAP), hereditary non-polyposis colorectal cancer, hereditary breast and ovarian cancer, multiple endocrine neoplasm], in which molecular mechanisms and clinical characteristics are well known [8, 9]. In addition to these well-recognised hereditary syndromes, there are some associations of MPM frequently observed (i.e., colon/stomach, breast/female genital system, endocrine/colon, and high respiratory system/upper gastrointestinal), which can represent an emerging field of research. Finally, there are very uncommon associations both for the rarity of each tumour involved and for the specific association between the two tumours.

In this paper, we focused on a few selected cases with rare tumours from our overall MPM series, and we made a comparison of these rare associations between our experience and data available from surveillance, epidemiology, and end results (SEER) cancer registries [7], the largest global series on this topic.

## Methods

From an overall series of 315 MPM-affected patients, who were observed at our department from 1980 to 2010, we selected six patients (1.9%) with uncommon MPM. In selecting these cases, we considered both the infrequency of each tumour type (e.g., oligodendroglioma, embryonal carcinoma of the testis, glioblastoma, liposarcoma of the thigh, and osteosarcoma) and the rarity of their associations in the context of MPM. We included in this analysis only those patients who were surgically treated for both tumours.

Patients with inherited syndromes were excluded. The patients’ characteristics (age and sex), the first and second tumours’ characteristics (site, TNM stage, and treatment), and the interval between the tumours have been analysed; finally, we compared our data to those available from SEER cancer registries [7], the largest global series on this topic.

## Results and discussion

**Figure 1.**
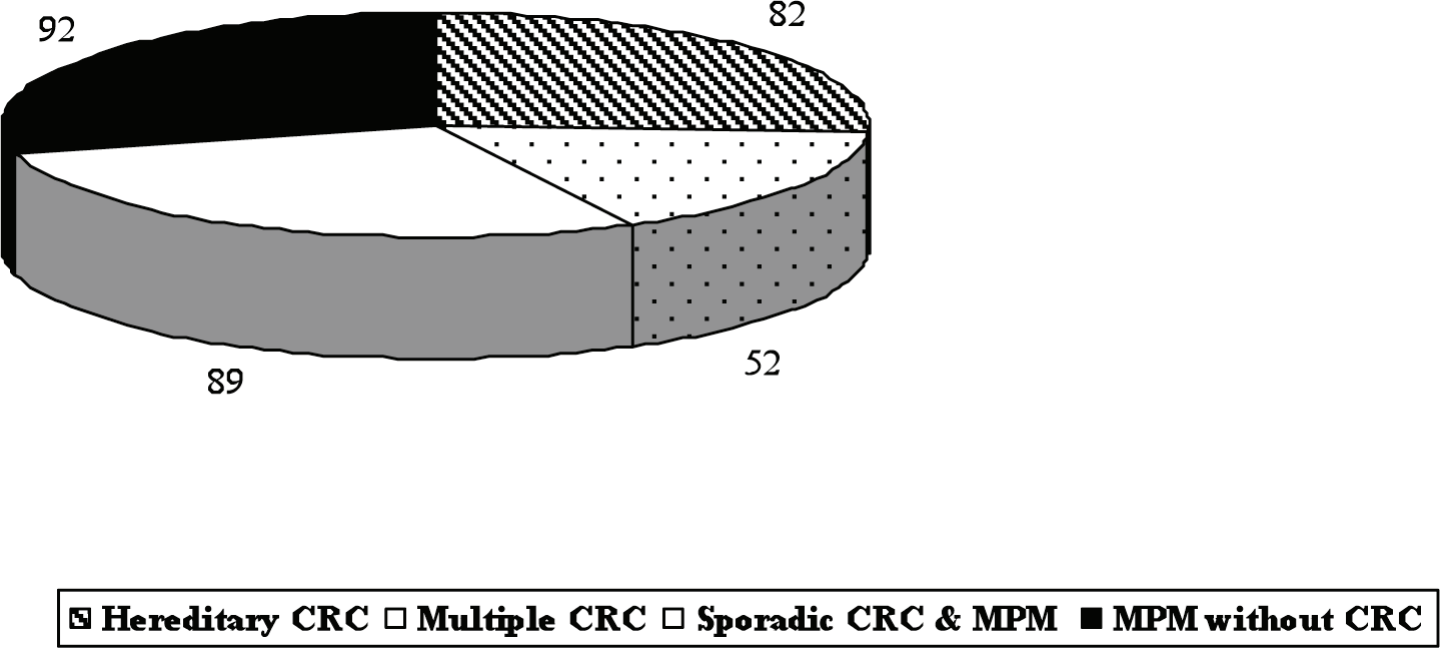
MPM: our overall series (315 patients, period 1985–2010).

Due to our special interest in gastrointestinal and inherited diseases [10–18], 223 patients (70.8%) with MPM had colorectal cancer (CRC): 82 hereditary and 141 sporadic CRC (Figure 1). Ninety-two patients presented MPM with several neoplastic associations excluding CRC. However, we decided to focus on those patients with uncommon tumour associations both for the rarity of each tumour type involved and for the specific association between the two tumours. Based on these criteria, we selected six patients who were surgically treated for both tumours.

In our selected population of six patients with uncommon MPMs, the first tumour was diagnosed at a median age of 36.5 years (range 23–58). The male:female ratio was 1:1. The first tumours were the following: oligodendroglioma (case 1), embrionic testicular tumour (case 2), glioblastoma (case 3), endometrial adenocarcinoma (case 4), adenocarcinoma of ascending colon (case 5), and osteosarcoma of the jaw (case 6). At diagnosis, none of these tumours was at an advanced stage of disease and all patients underwent curative surgery. In addition, five out of six patients received adjuvant chemotherapy (CT); two patients with brain tumour were treated with radiotherapy (RT) as well. At a median follow-up of 11.3 years (range 1–36 years) a second tumour was diagnosed: adenocarcinoma of ascending colon (case 1), gastric adenocarcinoma (case 2), clear cell renal carcinoma (case 3), soft tissue liposarcoma of the of thigh (case 4), glioblastoma (case 5), and breast cancer (cancer 6). All patients underwent surgery of the second tumour, namely, right colectomy (case 1), gastrectomy (case 2), nefrectomy (case 3), excision of liposarcoma (case 4), exeresis of glioblastoma (case 5), and mastectomy (case 6). The patients’ characteristics are detailed in Table 1.

The incidence of MPM is constantly increasing and is expected to be even higher in the coming years. It will depend on several factors, such as the increased cancer incidence itself, the overall longer lifetime, the improvements in adjuvant treatments with accordingly longer disease-free interval, the accuracy of follow-up in oncologic patients, and the quite good prognosis of various tumours [1–5].

The observation of patients affected by rare neoplastic associations gave us the opportunity to study further some aspects related to epidemiology, etiology, and clinics in patients with MPM and to compare our own data to those from SEER [7].

Soft tissue, bone, CNS, and testicular tumours are quite uncommon and represent 0.2–1.3% of all new cancer cases in the general population and 0.2–2.2% of cancer deaths [19–24]. However, patients recovering from these tumours have a risk of 2.5–14.1% of developing a second cancer [7, 20–22]. In our series, we observed tumour types associations different from those more frequently reported in the literature (Table 2).

With respect to the histopathology of the first CNS cancer (cases 1 and 3), the excess absolute risks were highest among people first diagnosed with medulloblastoma or ependymoma; these tumours tend to be diagnosed at a younger age and have better-than-average survival, and they are often treated with RT. Intermediate excess absolute risks were observed for new cancers following an initial astrocytoma, oligodendroglioma (our case 1), or mixed glioma. New malignancies occurred less often than expected following glioblastoma (our case 3) and malignant meningioma.

The most frequent sites of second cancers after a CNS tumour are lung, breast, and haematopoietic system. Subsequent CRC occurred more often than expected among people with mixed glioma (case 1), while the association between CNS and renal cancer (case 3) is less common (2.4%).

Testicular tumours (case 2) have generally good prognosis and high survival rates so the event of a second cancer is not negligible. Furthermore, the treatment of this disease may be potentially carcinogenic. Among men with seminoma, a significantly elevated risk was seen for leukaemia and new malignancies of the oesophagus, rectum, pancreas, and bladder, and for the combination of renal pelvis, ureters, and other urinary sites. Those with non-seminoma had increased risk for leukaemia and cancers of the oral cavity and kidney. In an international survey [25], the overall risk of developing a second cancer was similar for seminoma and non-seminoma, and both tumour types showed elevated risk for melanoma as well as cancers of the stomach, bladder, thyroid, and soft tissue. The association between testis and gastric cancer, observed in our study, has been reported in SEER in 1.3%.

Osteosarcoma generally shows an increased risk for acute non-lymphocytic leukaemia (ANLL) and for lung, bone, and soft tissue cancers, but in women most of the reports refer to breast cancer, as in our case 6.

In the other two patients (cases 4 and 5) the first tumours [uterine corpus cancer (UCC) and CRC] are among the most frequently observed in the general population, while their second cancers (liposarcoma and glioblastoma), which occurred in these patients, were among the less common and predictable.

The cumulative incidence of developing a second cancer following UCC [26] has been estimated by SEER for 17.5% at 25 years. The risk of developing a new malignancy (including second, third, and fourth cancers), however, may vary significantly by age at diagnosis, race, and subsequent cancer site although not substantially by histological type of UCC. There is significantly increased risk for subsequent tumours of the small intestine, colon, bladder, breast, vagina, and soft tissues, as well as acute leukaemia. Although the basic mechanisms of these associations in patients with neither Li-Fraumeni syndrome nor breast cancer susceptibility gene (BRCA) 1–2 mutation are not clear, it has been shown that endometrial, ovarian, and breast may have similar reproductive and menstrual risk factors [27, 28]. Hormonal factors may also play a role in colon cancer. However, while hormone replacement therapy increases the risk of breast cancer and UCC, it seems to have a protective role in colon cancer [29]. In addition, obesity and physical inactivity increase the risk of UCC, colon cancer, and post-menopausal breast cancer [20, 23, 30–32].

After CRC, there is an overall 7% increased risk of developing a new primary cancer, but the risk diminishes to levels prevailing in the general population when subsequent cancers of the colon, rectum, and anus are excluded [33]. Many new malignancies after CRC occurred in the digestive tract and may be related to shared lifestyle factors, including diet, obesity, and physical inactivity [34, 35]. The effect of these risk factors on hormonal metabolism, along with genetic susceptibility [20, 35], may explain the elevated risk of UCC seen among younger (ages <60 years) women diagnosed with CRC, as well as the increased risk of CRC among younger women with UCC. Obesity may also contribute to the elevated risk of kidney cancer after CRC [36]. However, the risk is limited to the first five years of follow-up with no increase in risk of CRC observed after kidney cancer, suggesting that medical surveillance may have played a role.

The associations (UCC/sarcoma and CRC/glioblastoma) we found are not frequent: SEER have reported just 11 soft tissue sarcomas as second tumour among 803 UCC (1.3%) and 52 glioblatomas out of 8791 CRC (0.6%). It has to be considered that a clear distinction between hereditary and sporadic CRC is not specified, so it is likely that many patients could belong to FAP affected families and these rates could consequently be even lower.

RT could be responsible for second tumours after UCC and CNS tumours. It seems that RT contributed to the elevated risk observed for subsequent ANLL and cancers of the rectum, urinary bladder, bone, and soft tissue, particularly because the excesses were noted primarily in women who received RT in the (neo)adjuvant setting [37]. For example, a large majority of new soft tissue and bone sarcomas diagnosed among UCC survivors receiving initial RT occurred in the irradiated pelvic fields. The risk for soft tissue sarcomas is increased by RT and possibly CT for other malignant conditions.

For testicular tumours a late-onset risk affected several sites within or near the radiation field, including a twofold or greater increase in risk for cancers of the digestive tract.

In some cases involving a CNS cancer a genetic hypothesis can be ascribed. Some hereditary syndromes in fact include CNS tumours, such as Li-Fraumeni syndrome [38–40], neurofibromatosis type 1 or Turcot, and in these cases the genetic mutations responsible for neoplastic transformation are well known. The association of CNS tumours and CRC appears to be particularly interesting. The knowledge of a genetic mutation in these patients leads us to speculate that a similar mechanism could also be at the origin of these so-called ‘uncodified’ MPM, such as those we observed. Our cases did not belong to FAP-affected families. It is conceivable that heritable genetic mutations currently unknown might stay at the basis of these associations also.

With regards to the same associations of tumours selected in our analysis, SEER database reports an interval time between the first and the second tumour diagnosis being variable from few to several years (Table 3). The mean interval time in our series was 11.3 years. An interval longer than ten years has been observed in 50% of patients (cases 1, 2, and 5) in accordance to SEER for the association between testis and gastric cancer. In our patients with CNS tumours and CRC the long interval (12 years) was different from SEER, in which the majority of these cases can happen in the first ten years. Cases 3 and 4 with a shorter interval are in accordance to SEER, while breast cancer generally follows bone tumour after more than ten years.

It is very difficult to find a clear etiopathogenesis in many of these cases, especially when the second tumour has followed the first after several years. Over such a long time frame, a patient may have had contact with different carcinogenic factors, so it is just possible to make some hypotheses, Iatrogenic, and genetic factors could explain the origin of the tumours in our observed associations.

It is paramount to stress that every physician must consider the onset of new malignancies for each neoplasm even many years after first diagnosis.

## Conclusions

Nowadays, MPM represent a challenge not only for surgeons, but also for many other physicians, such as oncologists, radiotherapists, endoscopists, and genetists.

If we had 20-year follow-up in all cases of cancer, a quarter of these would develop a second cancer.

When a patient recovers from a tumour his follow-up must be very strict and the risk of second tumour and their frequent association as MPM must be considered for each neoplasia even many years after the first diagnosis.

Advancements in biomolecular knowledge might answer many interesting questions and shed light on the etiological mechanisms of these associations. Tumour registries, large study groups, and interdisciplinary cooperation must be strongly encouraged to reduce mortality related to MPM and to foresee their occurrence. It is our aim that sharing our own experience and publishing even a small series and infrequent cases may be relevant and could be useful in the improvement of knowledge.

## Conflicts of interest

The authors have no conflicts of interest (political, personal, religious, ideological, academic, intellectual, commercial, or any other) to declare in relation to this manuscript.

## Authors’ contributions

NC carried out acquisition, analysis, and interpretation of data, and drafted the manuscript.

MLS made substantial contributions to acquisition, analysis, and interpretation of data.

RM carried out acquisition of data.

AC carried out acquisition of data.

CD carried out acquisition of data.

AR conceived the study, and participated in its design and coordination, critically revised the study.

## Figures and Tables

**Table 1.** Patients characteristics.

		First cancer /Index tumour					Interval between first and second cancer	Second cancer			
Case	Sex	Age	Site – histology	Stage/ grading	Surgery	Adiuvant treatment		Age	Site – histology	Stage/ grading	Therapy
1	F	28	CNS – oligodendroglioma	Low grade	Excision	RT + CT	12	40	Right colon – adenocarcinoma	pT3N0M0	Right colectomy
2	M	33	Testis – embryonic cancer	T2N0M0 Stage 1B	Orchiectomy	CT	36	69	Stomach – adenocarcinoma	T2G1N0	Gastrectomy
3	M	53	CNS glioblastoma	Low grade	Excision	RT + CT	1	54	Kidney – renal carcinoma	T1BN0M0	Nefrectomy
4	F	40	Endometrium – adenocarcinoma	Stage I	Histeroannessiectomy	CT	2	42	Soft tissue – liposarcoma of thigh	Low grade	Excision
5	M	58	Right colon – adenocarcinoma	T3N0M0	Right colectomy	–	12	70	CNS – glioblastoma	Low grade	Excision
6	F	23	Bone – osteosarcoma of the jaw	NS	Excision	CT	5	28	Breast – ductal cancer	T2G1N0	Mastectomy

RT = radiotherapy, CT = chemotherapy.

**Table 2.** Likelihood of second tumours after brain, testis, uterus, colon, and bone primary tumours in [3, 7, 20–22].

Site of cancer	Individuals survived at least two months (*n*)	Individuals who developed a second cancer (*n*)	O/E	CI=	EAR per 10,000 person years	Cumulative incidence at 25 years (%)	Most frequent site for second tumours
Brain	29,361	496	1.11	1.01–1.21	4	2.5	Lung Prostate^°^ Breast^*^ CNS
Testis	14,984	803	1.62	1.51–1.74	21	14.1	Prostate Testis Lung
Uterus	74,185	8791	0.99	0.97–1.01	29	17.5	Colon Breast Lung
Colon	179,370	20847	1.07	1.05–1.08	13	15.2	Colon Lung Breats^°^
Bone	4807	223	1.24	1.08–1.41	13	8.6	Lung Prostate^°^ Breast^*^ Haematopoietic

O/E = ratio of observed to expected subsequent cancers,

CI = cumulative incidence,

EAR = excess absolute risk,

° male,

* female.

**Table 3.** Interval time between first and second tumours in ‘rare’ MPM as reported by SEER database [7].

Index tumour/second tumour	Observed cases (n)	Interval between tumours < 10 years (n)	Interval between tumours > 10 years (n)
CNS/colon	42	33	9
Testis/stomach	11	4	7
CNS/kidney	12	9	3
Uterus/soft tissues	52	34	18
Colon/CNS	152	119	33
Bone/breast	22 (♀)	8	14
